# Transnasal Evaporative Cooling in Out-of-Hospital Cardiac Arrest Patients to Initiate Hypothermia—A Substudy of the Target Temperature Management 2 (TTM2) Randomized Trial

**DOI:** 10.3390/jcm12237288

**Published:** 2023-11-24

**Authors:** Akil Awad, Emelie Dillenbeck, Josef Dankiewicz, Mattias Ringh, Sune Forsberg, Leif Svensson, Andreas Claesson, Jacob Hollenberg, Per Nordberg

**Affiliations:** 1 Department of Clinical Science and Education, Center for Resuscitation Science, Karolinska Institutet, Södersjukhuset, 11883 Stockholm, Sweden; akil.awad@ki.se (A.A.); emelie.dillenbeck@ki.se (E.D.); mattias.ringh@ki.se (M.R.); sune.forsberg@ki.se (S.F.); andreas.claesson@ki.se (A.C.); jacob.hollenberg@ki.se (J.H.); 2 Department of Clinical Sciences, Lund University, Skane University Hospital, Cardiology, 22385 Lund, Sweden; josef.dankiewicz@gmail.com; 3 Department of Medicine, Karolinska Institutet, Solna, 17177 Stockholm, Sweden; leif.svensson@ki.se; 4 Function Perioperative Medicine and Intensive Care, Karolinska University Hospital, 17176 Stockholm, Sweden

**Keywords:** out-of-hospital cardiac arrest, hypothermia, transnasal evaporative cooling

## Abstract

**Background:** In animal models, early initiation of therapeutic cooling, intra-arrest, or restored circulation has been shown to be neuroprotective shortly after cardiac arrest. We aimed to assess the feasibility and cooling efficacy of transnasal evaporative cooling, initiated as early as possible after hospital arrival in patients randomized to cooling in the TTM2 trial. **Methods:** This study took the form of a single-center (Södersjukhuset, Stockholm) substudy of the TTM2 trial (NCT02908308) comparing target temperature management (TTM) to 33 °C versus normothermia in OHCA. In patients randomized to TTM33 °C, transnasal evaporative cooling was applied as fast as possible. The primary objectives were the feasibility aspects of initiating cooling in different hospital locations (i.e., in the emergency department, coronary cathlab, intensive care unit (ICU), and during intrahospital transport) and its effectiveness (i.e., time to reach target temperature). Transnasal cooling was continued for two hours or until patients reached a core temperature of <34 °C. Cooling intervals were compared to participants at the same site who were randomized to hypothermia and treated at 33 °C but who for different reasons did not receive transnasal evaporative cooling. **Results:** From October 2018 to January 2020, 32 patients were recruited, of which 17 were randomized to the TTM33. Among them, 10 patients (8 men, median age 69 years) received transnasal evaporative cooling prior to surface systemic cooling in the ICU. In three patients, cooling was started in the emergency department; in two patients, it was started in the coronary cathlab, and in five patients, it was started in the ICU, of which three patients were subsequently transported to the coronary cathlab or to perform a CT scan. The median time to initiate transnasal cooling from randomization was 9 min (range: 5 to 39 min). The median time from randomization to a core body temperature of 34 °C was 120 min (range 60 to 334) compared to 178 min among those in the TTM33 group that did not receive TNEC and to 33 °C 230 min (range: 152 to 351) vs. 276 min (range: 150 to 546). No feasibility or technical issues were reported. No adverse events occurred besides minor nosebleeds. **Conclusions:** The early induction of transnasal cooling in out-of-hospital cardiac arrest patients was feasible to initiate in the emergency department, coronary cathlab, ICU, and during intrahospital transport. Time to target temperature was shortened compared to standard cooling.

## 1. Introduction

Out-of-hospital cardiac arrest is a major health concerns, with approximately 300,000 people in Europe affected each year [1]. Despite decades of efforts to promote cardiopulmonary resuscitation, education, and the introduction of automated external defibrillators, less than 50% of cardiac arrest victims achieve a return of spontaneous circulation (ROSC), and this percentage drops to 20% or less for those patients that live in rural areas or those with an initial non-shockable rhythm (i.e., asystole and pulseless electric activity) [2].

Post-anoxic severe brain injury is the primary cause of death in resuscitated cardiac arrest patients, but despite decades of post resuscitation research, there are few, if any, evidence-based strategies currently available to improve neurologic outcome [3]. Thus, there is an urgent need to develop strategies to mitigate these injuries to improve neurologic outcomes after cardiac arrest.

Induced hypothermia (known more generally as targeted temperature management [TTM]) reduces ischemia-reperfusion brain injury in experimental models and may have the potential to limit the brain injuries in patients resuscitated from a cardiac arrest [4]. Animal data demonstrate a benefit of ultra-early cooling, initiated during CPR (i.e., intra-arrest) compared to cooling started at a later stage [5,6]. Despite this knowledge, the vast majority of clinical studies have assessed the effect of induced hypothermia or TTM to 33 °C (TTM33) initiated after hospital arrival, often hours after the arrest, with a substantial delay until the target temperature level has been reached [7,8]. Thus, this approach may have led to a failure to address the underlying pathophysiology of acute ischemia-reperfusion. Although the initial smaller randomized controlled trials with a highly selected cardiac arrest population (i.e., younger population, only witnessed arrest with shockable rhythms) showed benefit in neurologic outcomes from therapeutic hypothermia initiated after hospital arrival, the TTM2 trial with a more heterogenous patient group and some subsequent meta analyses did not show any difference in 6-month survival [8,9,10]. In addition, a recent, moderately sized randomized controlled trial showed the benefit of therapeutic hypothermia in patients with non-shockable rhythms, and a recent Cochrane review concluded that induced therapeutic hypothermia may improve neurologic outcomes [11,12].

The effect of early, prehospital cooling has been assessed in randomized studies, but the effect of this intervention on neurologic outcomes has been difficult to evaluate as the cooling modality itself (i.e., large amounts of cold fluids intra-arrest or shortly after ROSC) is associated with hemodynamic adverse events, metabolic derangement, and an increased risk of a new cardiac arrest prior to hospital arrival [13,14].

Transnasal evaporative cooling (i.e., RhinoChill^®^, BrainCool AB, Lund, Sweden) is a cooling method that can be used to induce intra-arrest cooling at the scene of the arrest, and the method has been shown to be safe and feasible in the prehospital setting. It has also been shown to shorten the time to target temperature in two randomized trials with a total of 877 out-of-hospital cardiac arrest patients [15,16]. The method has several advantages compared to cold fluid cooling as it cools effectively with continuous cooling without the need to administer fluids and load to the heart.

Although the method has been thoroughly studied in the prehospital setting, little is known about the feasibility of transnasal evaporative cooling in the hospital setting and during intrahospital transport between the emergency department, radiology department, coronary cath lab, and the intensive care unit. In current clinical practice, more diagnostics and in-hospital measures are performed prior to admission to the intensive care unit, which may delay the initiation of cooling even further without a suitable cooling method available that can be used during transport. Our hypothesis was that transnasal evaporative cooling could be applied to initiate cooling and would be as effective as standard intravenous or surface cooling at the intensive care unit to reach the target temperature. Thus, the aims of this study were to assess the feasibility of initiating transnasal evaporative cooling shortly after hospital arrival, such as in the emergency department, and to assess cooling efficacy (measured as time to target core body temperature) in out-of-hospital cardiac arrest patients randomized to therapeutic hypothermia in the TTM2 study.

## 2. Materials and Methods

### 2.1. Study Design and Ethics

This was an open-label substudy of the TTM2 trial (NCT02908308), wherein neither the healthcare staff nor the patients were blinded to the treatment. The TTM2 trial is an international multicenter randomized clinical trial that compared TTM33 °C (induction—maintenance—controlled rewarming) versus normothermia in the intensive care unit in out-of-hospital cardiac arrest patients [17]. In both study groups, fever defined as core body temperature < 37.8 °C was avoided for 72 h [8]. The primary outcome in the TTM2 trial was death from any cause at 6 months.

This single-center substudy was conducted at Södersjukhuset, a regional hospital in the greater Stockholm region; patients were enrolled between October 2018 and January 2020. This was primarily a feasibility study in which patients randomized to TTM33 °C received transnasal evaporative cooling prior to arrival at the intensive care unit and presystemic hypothermia with surface or intravascular cooling devices was initiated. The TTM2 trial was approved on the 24 September 2015 by the Regional Ethics committee (identification number 2015/228). Written informed consent was waived, deferred, or obtained from a legal surrogate, depending on the circumstances, and was obtained from each patient who regained their mental capacity.

### 2.2. Patients

The TTM2 trial included unconscious out-of-hospital cardiac arrest patients who had been admitted to the hospital with presumed cardiac issues or those of an unknown etiology, regardless of initial rhythm, with ROSC for at least 20 consecutive minutes. Exclusion criteria included unwitnessed cardiac arrest with asystole, limitations in care, or more than 180 min from ROSC to randomization. A detailed list of the inclusion and exclusion criteria has been previously described in [8]. Eligible patients were randomized in a 1:1 ratio, via the use of a web-based system, to undergo hypothermia or normothermia. Patients included in the TTM2 trial and allocated to 33 °C at Södersjukhuset were included in this substudy, where the only added exclusion criterion compared to the main trial was if patients had an obvious anatomic barrier to the placement of transnasal catheters.

Due to the inherent difficulty of concealing the allocation to TTM in the main trial, the treatment with Transnasal evaporative cooling treatment, and the patient’s body temperature, the healthcare staff caring for the patient were not blinded to the intervention.

### 2.3. The Transnasal Evaporative Cooling Method

**Figure 1:**
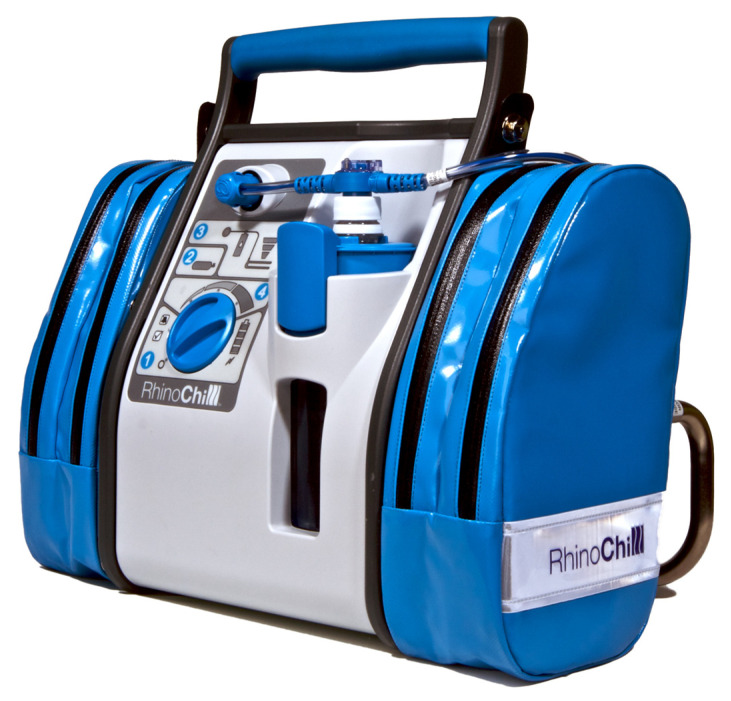
The transnasal evaporative cooling device (RhinoChill).

The currently available and CE-marked transnasal evaporative cooling system (RhinoChill^®^) is fully portable, weighs approximately 12 kg, and can be carried and hanged on the patient bed/stretcher (Figure 1). The device is battery-operated, and the battery lasts for about 3 h. When arriving at the coronary cath lab or the radiology department, one can disconnect the portable oxygen cylinder and connect to the hospital general oxygen supply. Transnasal evaporative cooling delivers an evaporating liquid coolant oxygen mixture into the nasal cavity through two nasal catheters. The liquid coolant, perfluorohexacene, is an inert and highly volatile chemical that evaporates immediately and thereby removes heat from surrounding tissues without adding any intravascular volume. The proximity of the nasal cavity to the brain enables the transnasal evaporative cooling method to act as a heat exchanger at the base of the skull and more selectively cool the brain. The cooling method can be initiated within minutes from the collapse, has been shown to be safe for use in out-of-hospital cardiac arrest, and has been described in more detail previously [15,16].

### 2.4. Study Intervention

After the in-hospital randomization, the intervention period of 40 h started. All patients in both study groups were sedated during the intervention period. In patients randomized to hypothermia, cooling was started as soon as possible with the target temperature of 33 °C. In the vast majority of patients, the randomization and initiation of cooling took place in the intensive care unit, and the median time to randomization was 136 min in the intervention group and 133 min in the control group. The core temperature of 33 °C was maintained for 28 h after randomization, followed by slow rewarming to 37 °C. Thus, the time with a core temperature of 33 °C could differ slightly between patients depending on the time they took to reach target temperature. The median time to reach a core body temperature of <34 °C was 3 h after randomization. Apart from the TTM intervention and sedation strategy, the subjects received standard post-resuscitation care according to the local standards of the treating hospital. At 96 h after randomization or later, a physician who was unaware of the intervention assignments assessed whether the criteria for a likely poor neurologic outcome were present. All decisions about withdrawal of life-sustaining therapy were at the discretion of the treating physician (guided by the protocol).

In the main trial, the patients in the intervention group were randomized at a median of 136 min after the cardiac arrest [8]. In this substudy, the aim was to start the transnasal evaporative cooling treatment immediately after inclusion, preferably upon arriving at the hospital’s emergency department. Since the device is portable, this could be achieved almost anywhere in the hospital (e.g., in the emergency department, the coronary cath lab, or the intensive care unit). If the patient passed through the radiology department or the coronary cath lab on the way to the intensive care unit, transnasal evaporative cooling could be continued; if started in the emergency room or in the intensive care unit, it could be started in these locations. As soon as possible after the patient arrived in the intensive care unit, TTM using surface cooling was started. Transnasal evaporative cooling was used for a maximum of 120 min or until the patient’s core temperature reached 34 °C. After that, only surface cooling was used for the rest of the intervention period.

### 2.5. Outcomes

The primary outcome was whether transnasal evaporative cooling was feasible for in-hospital use before systemic cooling was started in the intensive care unit in terms of time to initiate cooling, the location of cooling initiation prior to intensive care, and technical issues to applying transnasal evaporative cooling outside of the intensive care unit.

Secondary outcomes included cooling intervals, i.e., time from the cardiac arrest and randomization to start of cooling, time to core body temperatures of 34 °C and 33 °C, and safety aspects presented as device-related and as associated with complication rate within the first seven days of admission and survival at 30 and 180 days.

### 2.6. Statistical Analyses

Descriptive statistics were used. Binary variables are presented as number and percentage. Continuous variables are presented as median and range. Detailed and descriptive data for each patient receiving transnasal evaporative cooling are presented separately. Due to a small number of patients, no comparative statistical analyses were performed. Data are presented as treated, meaning that the two groups that are compared herein were divided post hoc depending on which cooling strategy was used. Thus, no intentional division of the two groups occurred during the study, and the reason why some patients did not receive transnasal evaporative cooling was not recorded. The transnasal evaporative cooling group was defined based on whether the device was used for a minimum of two hours or until a core temperature of 34 °C was reached.

## 3. Results

**Figure 2:**
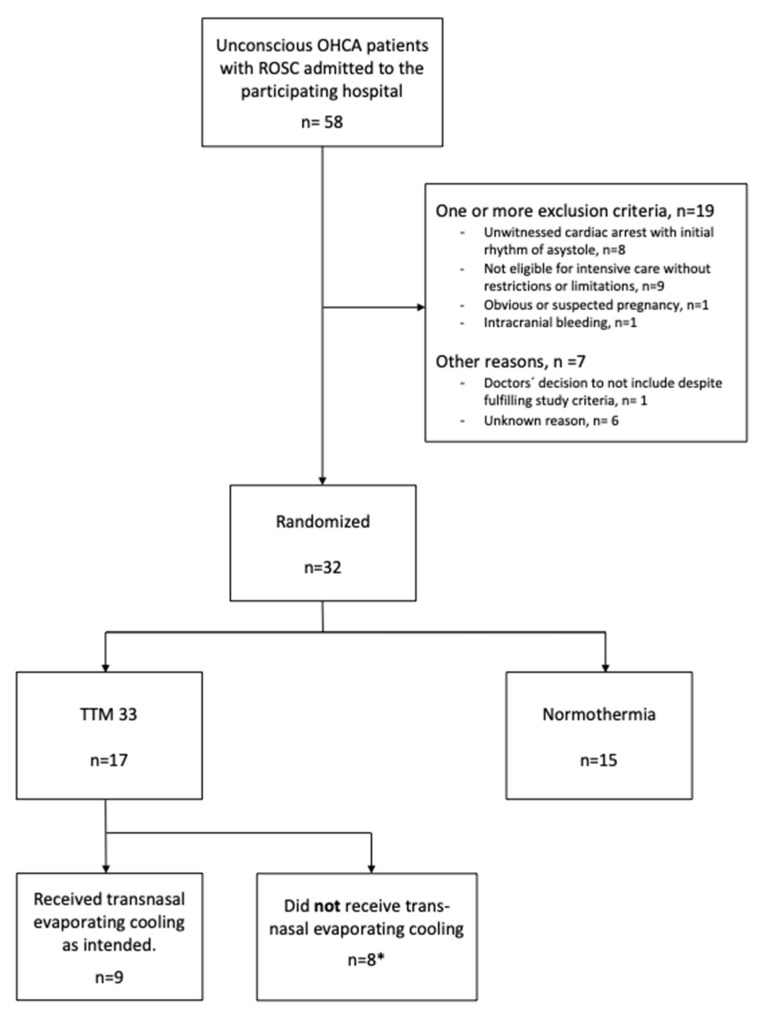
* In one patient, transnasal evaporative cooling was initiated for about one minute and then interrupted due to a risk of contagious disease; thus, this patient only received systemic surface cooling in the intensive care unit.

At the study hospital, 32 patients were included in TTM2, of which 17 were randomized to TTM at 33 °C. Among these, 10 patients received early cooling via transnasal evaporative cooling. In one patient, cooling was interrupted due to a patient with blood-borne disease having a nosebleed. The reason as to why some patients randomized to TTM33 did not receive transnasal evaporative cooling was not recorded but may have been due to various reasons, such as a lack of trained personnel at time of randomization, logistical reasons, or other circumstances. The patients admitted to the study hospital with sustained ROSC after cardiac arrest during the study period and the main reasons for exclusion are shown in Figure 2.

### 3.1. Baseline Characteristics

The baseline characteristics for the two different hypothermia groups are presented in Table 1 according to what strategy for induced hypothermia that was used. The median age was similar, as was the proportion of men in each group.

Among patients randomized to TTM33, patients that did not receive transnasal evaporative cooling, a higher proportion had heart failure and hypertension. In addition, this group had a higher proportion of shock—5/8 (63%)—compared to those receiving transnasal evaporative cooling—1/10 (10%)—prior to systemic cooling.

### 3.2. Feasibility

Among the 10 patients for which transnasal evaporative cooling was initiated, four treatments were initiated in the emergency room, one in the coronary catheter lab, and five shortly after arrival at the intensive care unit. In five patients, transnasal evaporative cooling was used in the coronary catheter lab. In four cases, the transnasal evaporative cooling continued as the patient was transported to the radiology department (see Figure 2). No technical issues regarding the use of the device were reported upon the initiation of its use, during intrahospital transfers, or at the different locations where it was used. An overview of cooling events and the devices’ trajectory with the patients receiving transnasal evaporative cooling are presented in Table 2.

### 3.3. Adverse Events and Safety

No major adverse events related to the device were reported. Three out of the ten patients had a minor nosebleed; in two of these cases, the nosebleed self-terminated within minutes and the treatment could continue as planned. One of the patients who experienced a nosebleed had a blood-borne infection; thus, the transnasal evaporative cooling treatment was terminated almost immediately (within minutes after initiation due to safety concerns for the medical staff). Two patients developed pneumonia, and one patient had sepsis during the first week. No other complications were reported. The results regarding survival at 30 and 180 days are presented in Table 3.

### 3.4. Cooling Intervals

The median tympanic temperatures at randomization were 36.4 (range 35.2–37.7) in the group where transnasal evaporative cooling was initiated vs. 35.0 (range 34.8–36.3) in the group treated with TTM33 but not receiving transnasal evaporative cooling. The median time from randomization to the start of transnasal evaporative cooling was 9 min. In patients receiving transnasal evaporative cooling, the median time from randomization to ≤34 °C was 120 min (at this time point, transnasal evaporative cooling was interrupted), the median from randomization to ≤33 °C was 230 min, the median time from ROSC to ≤34 °C was 186 min, and the median time from ROSC to ≤33 °C was 296 min (see Table 3). Patients randomized to TTM33 not receiving transnasal evaporative cooling had a median time from randomization to ≤34 °C of 178 min and had a median time from ROSC to ≤34 °C of 250 min. The cooling rate per hour (measured from randomization) in the intervention group receiving transnasal evaporative cooling was 1.2 °C per hour vs. 0.33 °C per hour among those receiving TTM33 without transnasal evaporative cooling.

## 4. Discussion

The main finding of this single-center feasibility substudy of the TTM2 trial was that transnasal evaporative cooling can be initiated in out-of-hospital cardiac arrest patients at a very early stage after arrival to hospital. The cooling treatment could be started very quickly, within minutes from randomization at the emergency department, coronary catheterization lab, or at the intensive care unit after the device had been brought to the site of randomization. Transnasal evaporative cooling was performed without any technical issues or significant safety concerns.

Experimental studies have repeatedly shown the importance of the early initiation of cooling after cardiac arrest, and it has been suggested that to achieve an optimal effect, cooling needs to be started intra-arrest or within 15–20 min from ROSC. However, this is difficult, and in clinical practice, TTM is most often applied late, often after several diagnostic and therapeutic measures that are performed prior to admission at the intensive care unit, such as a CT scan and/or coronary angiography with potential percutaneous coronary intervention. This may take several hours before the patient arrives at the intensive care unit, thus delaying the start of cooling. Although it is possible to initiate systemic surface cooling or intravascular cooling in the emergency department, both of these methods are considerably more complicated and time consuming, requiring urgent vascular cannulation from experienced physicians. One also needs to take into account the weight/dimensions of the surface and intravascular cooling devices and the fact that this type of systemic cooling, for practical and technical reasons, has to be interrupted during transport within the hospital. Thus, in general, there is a substantial delay of several hours until cooling can be initiated upon arrival at the intensive care unit, with subsequent delays until the target temperature level has been reached. Based on theoretical reasoning, this may, as discussed above, attenuate a possible effect of hypothermia as the time window for initiating TTM may be missed [11]. Thus, there is a need to identify patients that might benefit from TTM and to find ways to provide effective cooling at an earlier stage.

There are very few hypothermia studies that have assessed the period ranging from when the patient arrives at the hospital’s emergency department to the initiation of systemic cooling at the intensive care [18]. Taken the pathophysiological mechanisms into account, this time period, occurring shortly after ROSC, may be particularly important to mitigate ischemia-reperfusion injury. In addition, transferring patients to so-called cardiac arrest centers may also substantially delay the time to initiation of potentially neuroprotective strategies such as cooling if one cannot initiate and continue the treatment during transport. In a recent study assessing the early vs. late initiation of TTM, it was shown that the early initiation of TTM (Door-To-TTM) in out-of-hospital cardiac arrest patients was associated with increased survival and with better neurological outcomes among these patients [19].

Despite the limited number of patients in this study, this study shows that transnasal evaporative cooling is a method that can easily be induced in different settings within the hospital, such as in the emergency department, in the coronary cathlab, or in the intensive care unit prior to transportation to the coronary cathlab or for other diagnostic measures. We believe that this is important as other prehospital hypothermia trials have provided cooling in the prehospital context and lowered core body temperature but have subsequently failed to maintain the cooling effectively after hospital arrival. Thus, a gap in cooling may imply that the temperature increases after arrival at the emergency department and during in-hospital measures prior to when systemic cooling is started in the intensive care unit. This cooling/rewarming phenomena may be a critical factor when treating patients with therapeutic hypothermia after cardiac arrest. The method of transnasal evaporative cooling has previously been shown to be feasible and effective for lowering temperature in the prehospital setting when applied intra-arrest. The main advantage of the method is that it can be applied very early in the course of resuscitation to cool patients continuously, and it can be applied without the volume load that has been shown to be deleterious with cold fluids in the induction phase [20]. In this study, we observed that transnasal evaporative cooling cools at least at a similar rate as applying systemic cooling alone. Among patients receiving transnasal evaporative cooling, the cooling rate was 1.2 °C per hour, which is comparable with other systemic cooling devices. In the study, transnasal evaporative cooling was used until the patients had reached a core body temperature of 34° C or lower for a maximum of two hours. Thus, in this study, we did not study the effects of transnasal evaporative cooling on lowering the core body temperature from 34 to 33 °C.

In a previous hospital study where 84 OHCA patients had TNEC applied in the ED, the cooling rate of the cooling device was 1.1 °C per hour [21]. This is most likely similar to intravascular or surface cooling devices used in the intensive care unit. Thus, transnasal evaporative cooling may be an effective option for establishing and maintaining cooling in the prehospital setting or at an early stage after hospital arrival. In the PRINCESS trial, when cooling was applied intra-arrest at the scene of the arrest, a core body temperature of <34 °C could be reached after 105 min from the cardiac arrest, about 30 min before the median time to randomization in the TTM2 trial.

Among the patients receiving transnasal evaporative cooling, no serious adverse events were reported. The minor nose bleeds that occurred did not cause any prolonged problems, and in all patients’ cooling could be continued as planned. When one specific patient had a nosebleed, the cooling was stopped, as the patient had a contagious blood-borne disease. The adverse event rate in PRINCE and PRINCESS were similar to this study, with nosebleeds occurring in about 10 to 15% of the patients receiving transnasal evaporative cooling. In line with our findings, in the aforementioned trials, the nosebleeds most often resolved spontaneously within minutes and cooling could continue thereafter.

### Limitations

The main limitations of the present study were the limited number of study participants, its single-center setting, and the lack of data regarding the reasons for not initiating transnasal evaporative cooling. Among the patients randomized to TTM33, seven of them did not receive transnasal evaporative cooling. There may be several reasons for this. Firstly, most of these cases occurred early in the study period; thus, the lack of transnasal evaporative cooling may have been due to a lack of fully trained personnel at the time of randomization, taking the learning curve for the personnel at the emergency department and at the intensive care unit into account. Unfortunately, we did not collect data on why transnasal evaporative cooling was not started in these cases. Secondly, several of these cases occurred during the night, meaning that the lack of transnasal evaporative cooling may have been due to capacity problems and medical priorities when you have less personnel at the time of randomization. Thirdly, in two patients, TTM was not started due to circulatory instability, and one of them died just hours after inclusion. This may well have been a significant factor in the decision to provide transnasal evaporative cooling in these patients. Another limitation of this study was that the duration of transnasal cooling was set to two hours or until a core body temperature of 34 °C was reached. We could clearly see that the cooling rate was reduced when transnasal evaporative cooling was discontinued at a core body temperature of 34 °C. During the study period, there may have been instances wherein patients proved difficult to manage and/or instances characterized by a fear of overshooting (i.e., going too low in core body temperature) among personnel unexperienced with systemic cooling devices, causing delays in the patients reaching the target temperature of 33 °C.

In conclusion, early transnasal evaporative cooling using the RhinoChill system was found to be feasible to initiate in out-of-hospital cardiac arrest patients shortly after arrival at the hospital’s emergency department, at the coronary cath lab, at the intensive care unit, and before and during intrahospital transport. Time to core body target temperature was shortened compared to standard cooling. No significant technical or safety issues were observed.

## Figures and Tables

**Table 1:** Baseline Characteristics of patients randomized to hypothermia in the TTM2 trial receiving two strategies to induce hypothermia. Groups are divided as treated.

	Intervention: Transnasal Evaporative Cooling Prior to Surface Hypothermia (N = 9)	Intervention: Surface Hypo-Thermia with No Transnasal Evaporative Cooling (N = 8)
**Demographic characteristics**		
Age—median yr (range)	69 (54–89)	69 (56–77)
Male sex—no (%)	7 (78)	6 (75)
**Medical history**		
Hypertension—no (%)	3 (33)	6 (75)
Diabetes—no (%)	2 (22)	1 (13)
Myocardial infarction—no (%)	1 (11)	1 (13)
PCI—no (%)	1 (11)	1(13)
CABG—no (%)	0	0
Heart failure—no (%)	0	2 (25)
Level of fitness *—median (range)	3 (1–4)	2.5 (2–3)
**Characteristics of the cardiac arrest**		
Ventricular fibrillation—no (%)	4 (50)	4 (50)
Pulseless electrical activity—no (%)	2 (25)	2 (25)
Asystole	2 (25)	2 (25)
Location at home—no (%)	5 (56)	6 (75)
Bystander witnessed cardiac arrest -no (%)	8 (89)	8 (100)
Bystander CPR—no (%)	7 (78)	6 (75)
Median time from cardiac arrest to sustained ROSC—min (range)	25 (7–75)	20 (8–100)
Median time from cardiac arrest to randomization—min (range)	89 (43–188)	116 (39–248)
**Clinical characteristics on admission**		
Tympanic temperature—median °C (range)	36.4 (35.2–37.6)	35.0 (34.8–36.3)
Bilateral pupillary reflex present—no (%)	7 (78)	4 (100)
Arterial pH—median (range)	7.1 (7.0–7.4)	7.1 (6.6–7.4)
Arterial lactate level—median mmol/liter (range)	8.7 (1.3–14.3)	10.2 (2.9–19)
Shock—no (%)	1 (11)	5 (63)
ST-segment elevation myocardial infarction—no (%)	0	1 (13)

* Clinical Frailty Score, 1–9 (1 = very fit, 9 = terminally ill).

**Table 2:** Cooling events, passage through the hospital and safety for patients receiving TNEC *.

	Location of TNEC Initiation	Time from ROSC to Start of TNEC (min)	Time from Randomization to Start of TNEC (min)	TNEC Used in ER (Yes/No)	TNEC Used in CT/Radiology Lab (Yes/No)	TNEC Used in Cath-Lab (Yes/No)	TNEC Used in ICU (Yes/No)	Adverse Events
Patient # 1	ER	48	12	Yes	Yes	Yes	Yes	-
Patient # 2	Cath-lab	136	10	No	No	Yes	Yes	Minor nosebleed
Patient # 3	ICU	74	5	No	Yes	No **	Yes	-
Patient # 4	ER	50	5	Yes	Yes	Yes	Yes	-
Patient # 5	ICU	Not recorded	Not recorded	No	No	No **	Yes	-
Patient # 6	ER	43	9	Yes	Yes	No **	Yes	
Patient # 7	ICU	98	15	No	No	Yes	Yes	
Patient # 8	ICU	85	6	No	No	No **	Yes	
Patient # 9	ER	55	8	Yes	No **	Yes	Yes	Minor nosebleed

TNEC = Transnasal evaporative cooling. * Transnasal evaporative cooling. ** Was not performed.

**Table 3:** Outcomes of patients randomized to hypothermia in the TTM2 trial receiving two strategies to induce hypothermia. Groups are divided as treated.

	Intervention Receiving TNEC (N = 9)	Intervention Standard (N = 6) *
Time from ROSC to target temperature ≤ 34 °C—median (min)	186 (133–359)	250 (167–552)
Time from ROSC to target temperature ≤ 33 °C—median (min)	296 ** (218–480)	335 *** (258–612)
Time from randomization to target temperature ≤ 34 °C—median (min)	120 (60–334)	178 (98–486)
Time from randomization to target temperature ≤ 33 °C—median (min)	230 ** (152–351)	276 *** (150–546)
30-day survival—no (%)	6 (67)	2 (33)
180-day survival—no (%)	6 (67)	2 (33)

* Of patients randomized to intervention but did not receive TNEC, 6 of 8 patients started terapeutic hypothermia. ** 1 of 9 patients did not reach ≤33 °C. *** 1 of 6 patients did not reach ≤33 °C. TNEC = Transnasal evaporative cooling.

## Data Availability

The data are not publicly available in accordance with ethical approval and institutional regulations of patient data management.
